# The American Academy of Medicine

**Published:** 1886-01

**Authors:** 


### THE AMERICAN ACADEMY OF MEDICINE.

At the ninth annual session, held in New York City on November 28th and 29th, 1885, Dr. R. L. Sibbett, of Carlisle, Pa., read a paper on *The Study of Medicine as a Means of Education*. There are at present 87 "regular" medical colleges and 29 sectarian schools in the United States. In Canada 12 regular schools and none teaching sectarian medicine. Our colleges are too lax in their demands, both of matriculates and of candidates for graduation. A preliminary, thorough, academic course and systematic graded courses in medicine are earnestly recommended. Examinations by a State Medical Board of Examiners of applicants for license to practice, irrespective of the degrees they may hold, would be a very patent means of increasing the average grade of medical knowledge.

Dr. C. McIntyre, of Easton, Pa., read a paper on *Medical Supervision of Student Life.* The present great demands upon the mental energies of the student are detrimental to his bodily health and development. His suggestions were to remedy this in a measure by a careful physical examination at the beginning of school-life, and by a systematic graded series of gymnastic exercises, under medical supervision, during student-life.

Dr. Henry O. Marcy, of Boston, read a paper on *The Climatic Treatment of Disease,* with an illustration of western North Carolina as a health-resort.

The etiology of zymotic disease, based upon our knowledge of the rôle of bacteria, was first considered. He reviewed the recent investigation by climatologists of the mountain health-resorts of Europe and America and their effects, and concluded that the careful adaptation of the climate to the individual must be sought with reference to the improvement of his resisting vital powers, enabling him to withstand his bacterial enemies rather than to escape them.

The mountains of western North Carolina were described as offering a suitable climate for a variety of diseases, the writer's deduction being based upon a personal acquaintance with this region.

At the

EVENING SESSION

The President, Dr. Albert L. Gihon, delivered the annual address, the subject chosen being *What is Medicine?*

The Academy of Medicine is not a society of specialists. Its only limitation to fellowship is that the candidate shall be "learned in medicine," and in all else that the term implies—

a substantial foundation of thorough academic training for the study of medicine. This is one reason of the slow growth of the Academy. A second lies in its necessary hostility to men and institutions that defy the principles of its constitution. A third lies in the indifference of those who claim to be its friends.

In answer to the question, "What is Medicine?" we will find that it is the most profound and ennobling study that can engage the intellect of man. It embraces all that relates to the existence of man; his place in nature; his origin, development and growth; his preservation and continuance. The prevention and cure of disease is but a part, and to be mastered all else pertaining to it must be studied and comprehended. Only a well educated mind can accomplish the task. The proper place of the physician is not recognized in society. He is put on a level with the barber, cook, or cabman, by those who patronize him. Army and navy officers are jealous of his standing among them, and desire to prevent his promotion.

The low state of the American profession, in point of culture, was referred to with regret, and its cause found in the notorious looseness of those mercenary colleges whose practices shame our educational system.

Society should demand that no ignoramus be allowed a diploma.

Where a drug-clerk kills one patient by his carelessness, he saves a hundred by recognizing errors in doctors' prescriptions.

The debasement of medical education to the capacity of the purchaser of a diploma, will react upon the honor and reputation of every member of the profession. It must not go on.

To change all this, reforms must take hold of existing methods, with the deliberate intention radically to alter them.

Preliminary education must be the starting point. By insisting upon this other improvements would be secured correlatively.

SECOND DAY.

A paper by Dr. T. J. Turner, of the U. S. Navy, on *Medical Evidence* was presented. He said, expert-testimony differs from that given by other witnesses in that "matters of opinion" as well as "matters of fact" are admissible. The boundary-line separating expert from ordinary testimony may, however, be a matter of dispute. A jury may accept anything as expert-testimony which it sees fit. As a matter of fact, the writer said, "It has been decided that a medical opinion may be received as evidence if it is based upon study without practice, or upon practice without study, and it has been ruled that it is not absolutely necessary that the expert should have either studied or practiced medicine. In the place of "expert-testimony" the writer preferred the term "opinion evidence." The question of the admissibility of such evidence was to be decided by professional skill, attainments, aptitude, education, experience and known opportunities for observation of the subject on which the expert-opinion is demanded.

Drs. R. J. Dunglison, of Philadelphia, and H. O. Marcy, of Boston, made their *Report on Laws Regulating the Practice of Medicine in the United States and Canada.*

Indiana and North Carolina are the only states in which new and better laws have been passed within the year.

The New York law of 1884 had done some good.

Massachusetts had no law of any value.

In Pennsylvania the law had stopped non-graduates from practice.

The Michigan law proved weak.

In Ohio it was better but more legislation was wanted.

Tennessee had no law.

Wisconsin had a new law.

Kentucky had a law requiring a diploma.

Dakota had established a Territorial Board-of-Health, which granted licenses.

Texas is without legislation on the subject.

In New Jersey the registration law is weak.

Nebraska has registration.

Iowa has no statutes on the subject.

Virginia has had for two years a law which works fairly well.

Maine has no law.

New Hampshire has weak ones.

In Maryland the same is true as in New Hampshire.

Much remains to be done before the proper legal restrictions shall have been placed upon the practice of medicine, in all parts of the country. Progress has been slow, but there is no reason to despair.

Dr. Benjamin Lee, of Philadelphia, read a paper on *Health-Officers, Ancient and Modern.* In ancient Rome officers of the public health received high honors and emoluments, and Rome was a healthful city in those days.

Boards-of-health should be carefully chosen in reference to qualifications, not political but professional.

Physicians should compose such boards for the most part, but not wholly, for the addition of a man-of-affairs was necessary to make up for the well-known lack of business ability among physicians.

The municipal government should be represented upon the board-of-health to promote harmonious action.

Paid agents should be regularly employed by the board, and all the members thereof should receive some salary in order to secure efficient service.

Dr. Nelson read a paper on *Micro-organisms, and their Relation to Disease.* The natural history of these small organisms was now being studied, not merely with reference to pathogeny and prophylaxis of virulent diseases, but also as tending to throw light on such questions as "spontaneous generation," the causes of fermentation, and the deeper mysteries surrounding the origin and propagation of life. Bacteria, which are vegetable forms, are best classified as (1) bacilli, (2) micrococci. Their examination is difficult, and requires the best lenses and condensers. Even then their life-history must be followed out in order to differentiate one from another, they having nearly dentical forms at any single stage.

The theory of the causation of diseases of the infectious type, by bacteria, was gaining acceptation. In reference to some it was proven.

Dr. Nelson has investigated this subject both in Germany—under Koch—and in his own laboratory and speaks with some authority, therefore.

Dr. Cushing, of Boston, read a paper on *Observations Concerning the Relation of Bacteria to Certain Puerperal Inflammations.* The results of examinations of the bodies of women, who died in Vienna of puerperal fever, were here explained. Strepto-bacteria and strapleglo-coccus and in cases the bacillus pyogenes fœtibus were found. The strepto-coccus is not distinguishable from erysipelas bacilli, the cocci being in pairs and each pair forming a link.

In pelvic abscesses these organisms appear in the lungs and joints.

Puerperal inflammations resemble infected wounds, and treatment should be based upon this idea of the pathology.

Dr. R. S. Sutton, of Pittsburg, said that in abdominal surgery it was not germs from the air but from the fingers, instruments, etc., which set up mischief. The best results had been attained without antiseptics, so far, and that chemical agents caused irritation to the peritoneum.

Dr. H. O. Marcy, of Boston, related a case wherein a child had a suppurating peritoneal cavity which contained streptococci. The pus was evacuated and the cavity washed out with a solution of corrosive sublimate, after which the case recovered.

Dr. Jackson read a paper on *Medical Licenses and Medical Honors.* The great increase in medical colleges was noted, and the fact alleged to show that more practitioners were now college-graduates than formerly. It was only in 1881 that the diploma became a license to practice, and this rendered competition inevitable among colleges for the patronage of those who wished to purchase the legal right to practice.

The following were read by title:

Dr. Kelly. *The Physician and his Patient.*

Dr. Bush. *The Physicians of Delaware in the Eighteenth Century.*

